# Influence of a sport-oriented exercise concept on motor performance and sustainability in healthy but sporting inactive older adults aged 60+

**DOI:** 10.3389/fspor.2026.1702331

**Published:** 2026-03-25

**Authors:** Anneke Schumacher, Marlene Krumpolt, Lucas Sannemann, Kerstin Witte

**Affiliations:** Department of Sport Science, Otto-von-Guericke-University Magdeburg, Magdeburg, Germany

**Keywords:** healthy aging, low-threshold sport programs, motor performance, movement promotion, psychosocial well-being

## Abstract

Physical inactivity in older adults remains a public health concern despite the well-documented benefits of exercise for physical, cognitive, and psychosocial health. Many programs fail to reach inactive individuals, particularly men over 60, and often lack sustainability. Therefore, a low-threshold concept was developed that integrates strength, endurance, and coordination training while particularly emphasizing a variety of popular sports activities. This non-randomized controlled study evaluated the influence on selected motor outcomes and sustainability of this concept for healthy but previously inactive adults aged 60+, focusing on motor performance, psychosocial well-being, and long-term adherence. 161 older adults (68.23 ± 4.47 years, 66% female) who were healthy but previously sporting inactive (no regular weekly sports participation) completed a 24-week exercise program with two weekly 90-minute sessions combining multicomponent fitness training and introductory classes to recreational sports (e.g., karate, table tennis, handball, archery). Assessments were carried out at six measurement points: at baseline (t0), intermediate (t1), and post-intervention (t2), with follow-up surveys at 4 weeks, 6 months, and 12 months. Motor abilities were assessed for maximal grip strength, strength capacity of the upper and lower extremities, motor reaction ability, lower body flexibility, and cardiovascular fitness. Motives of sport, psychosocial well-being and sustainability were assessed via questionnaires. Nonparametric Brunner–Langer model, a mixed ANOVA and *post hoc* tests were used to compare intervention and control group (*n* = 32; 65.63 ± 3.63 years, 69% female) across the measurement time points t0–t2. Significant improvements with moderate effects for time*group were found in lower-limb strength [30s-chair-stand: *χ*^2^(1.84) = 5.127, *p* < .001, *Δ*RTE = .150] and cardiovascular fitness for within-group effects (PWC130: *p* = .017, ƒ = .25), and also small within-group effects of the intervention group for muscular endurance (biceps curl: *p* < .001, r = .06), motor reaction (drop-bar: *p* = .040, r = .03), flexibility (sit-&-reach: r = .03), with sex-specific advantages for women in endurance gains. Participants reported higher subjective well-being, increased social activity (40%) and positive feedback from their social environment. Sustainability was high: 97% of participants continued regular training after the intervention, 77% joined local sports clubs, and after 12 months, this proportion increased to 87% membership. The low-threshold, socially embedded, and gender-sensitive exercise program improve functional fitness, psychosocial well-being, and long-term adherence in previously inactive older adults, supporting active aging and providing a model for scalable community-based interventions.

## 1 Introduction

The demographic shift and the resulting increase in the proportion of older age groups pose new challenges for health promotion and prevention. Although contemporary perceptions of aging portray older adults as healthier, more active, and more mobile than previous generations, a substantial share of adults in Germany remains physically inactive. Despite the well-documented positive effects of physical activity and exercise on health, quality of life, and functional independence, only approximately 42.2% of older adults meet the WHO's physical activity recommendations (1–4). For many older adults who have been inactive for years, physical inactivity may appear more attractive than engaging in sport. The reasons for this often lie in a lack of motivation, psychosocial barriers - particularly among beginners or those returning to activity - or a shortage of suitable sports programs (5–7). Existing health-oriented exercise programs predominantly reach individuals who are already active, rarely succeed in motivating men over the age of 60 to participate, and generally fail to facilitate a sustainable transition into regular physical activity (8–14).

### 1.1 Evidence on physical activity in older age

Numerous studies demonstrate that age-related declines in strength, endurance, and coordination are not solely attributable to the natural aging process but are largely the result of physical inactivity (15, 16). Regular physical activity exerts preventive effects against chronic diseases, preserves cognitive and physical functions, and reduces the risk of requiring long-term care (17, 18). Moderate physical activity of 120 min per week yields marked differences in motor performance, such as strength of the upper and lower extremities, balance, flexibility, or endurance, between active and inactive older adults (19, 20). Physically active adults over the age of 60 can achieve performance levels comparable to those of inactive individuals aged 20–30 years (20). Furthermore, recreational and leisure sports such as dancing, karate, or tai chi - due to their synergistic interaction of various movement components - can simultaneously train and enhance multiple motor and cognitive functions in older adults, in contrast to purely strength- or endurance-focused exercise interventions (21–23). Physical activity includes not only structured exercise and sports but also everyday movements such as walking, cycling for transportation, housework, and gardening, all of which contribute to energy expenditure and health in older adults. Beyond motor benefits, it also promotes psychosocial health and psychological well-being in later life (24). In particular, engaging in sports within a group setting fosters social integration and group dynamics, thereby enhancing older adults’ sense of belonging, self-confidence, quality of life, and resilience (5, 25–27).

### 1.2 Concepts for promoting physical activity in Germany

In Germany, numerous initiatives exist to promote physical activity among older adults, such as “Fit im Alter”, “IN FORM”, rehabilitation sport, and programs offered by health insurance providers (1, 28). These initiatives typically focus on maintaining health-related functions (e.g., fall prevention) but seldom incorporate performance-oriented or recreational sport elements, and thus have limited reach among physically inactive target groups. In comparison, initiatives such as ‘walking football’ - a slower-paced, joint-friendly variant of soccer that offers promising opportunities for promoting healthy ageing - have recently emerged in some Europe countries (24, 29).

A key challenge lies in the scarcity of systematically evaluated programs with demonstrated sustainability and long-term continuation (10). In many cases, there is not only a lack of long-term evaluation and structural integration into local organizations (e.g., sports clubs) but also insufficient embedding within municipal structures. Although recommendations for sustainable physical activity promotion are available (18, 28, 30), a considerable proportion remain at the pilot-project stage. Furthermore, gender-specific motivational factors are often neglected in program design and outreach (12, 27). While behavioral changes in the domain of physical activity can be achieved in the short term, they are rarely sustained and permanently implemented in Germany (31). It can be assumed that behavioral and structural prevention measures will only have a significant effect if they are low-threshold, locally accessible, and socially anchored (18).

Against this background, there is a need to evaluate approaches that go beyond traditional preventive measures and provide accessible, socially embedded opportunities for physical activity. The present study aims to examine the influence of a recreational, sport-oriented physical activity program designed to facilitate both first-time entry and re-entry into sports among healthy but sporting inactive adults aged 60 years and above, regardless of prior sports experience. Specifically, the study investigates (i) the program's influence on selected aspects of motor performance, (ii) whether training-related adaptations differ between men and women, and (iii) indicators of long-term sustainability of physical activity participation. Given that physically inactive men over 60 are particularly difficult to reach with conventional health-oriented exercise offers, the program therefore placed specific emphasis on addressing this group and systematically examining sex-specific differences in training adaptations and long-term participation. In addition, potential benefits for psychosocial well-being are explored. Existing interim findings on program sustainability (7) are complemented and substantiated using additional follow-up data. The positive effects on cardiovascular fitness reported in a previous study (32) are now corroborated by an analysis including a control group.

## 2 Methods

### 2.1 Study design

The present study was designed as a 24-week intervention study, employing a pre-post design with an intervention group (IG) and an inactive control group (CG), conducted from February 2022 to December 2024. The physical activity promotion program for healthy but sporting inactive older adults was conceived as a low-threshold offer to encourage new or renewed engagement in sports.

**Figure 1:**
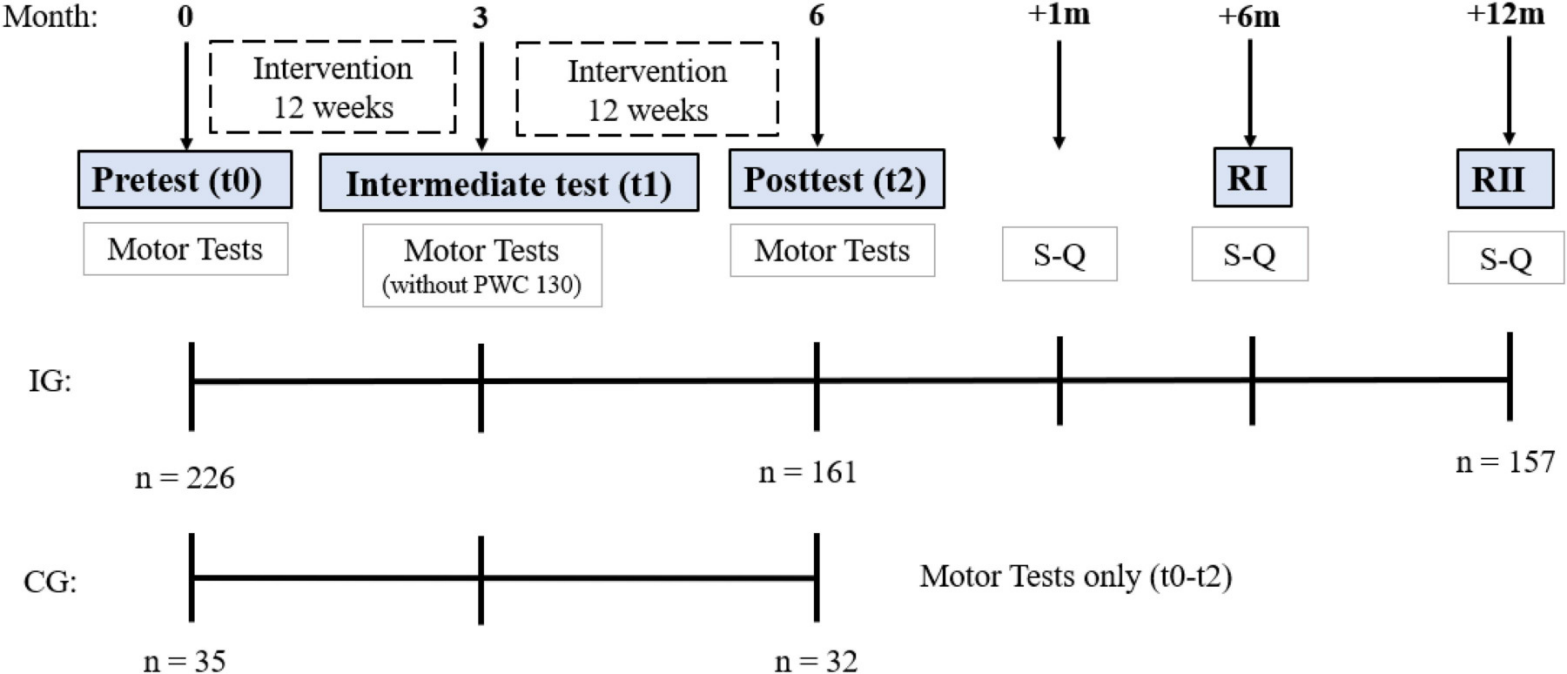
Timeline of the 24-week intervention period with assessment time points. Motor performance tests (hand dynamometer test, biceps-curl-test, 30s-chair-stand-test, drop-bar, sit-and-reach-test, PWC130) were conducted for both intervention group (IG) and control group (CG) at baseline (t0), after 12 weeks (t1), and after 24 weeks (t2). Sustainability Questionnaire **(S,Q)** was completed by IG participants 4 weeks post-intervention; Retention tests I (RI) and II (RII) followed at 6 and 12 months. Sample sizes indicate completed assessments.

Motor performance measurements and questionnaire surveys were conducted at three time points: prior to the start of the training intervention (t0), after 12 weeks of intervention (t1), and at the conclusion of the exercise program immediately after the intervention period (t2). In addition, further questionnaire-based data collection took place four weeks after the observation period (Sustainability-Questionnaire; S-Q), six months (Retention test I; RI), and up to one year after participation (Retention test II, RII). The CG received no intervention but underwent the same assessments as the intervention group (t0–t2; see Figure 1).

### 2.2 Sample description

A total of 226 older adults (148 female and 78 male) were recruited through local newspaper advertisements and flyers for the pre-test assessment (t0) in the intervention group (IG). Participants for the CG (*n* = 35; 24 female and 11 male) were recruited via additional local advertisements and received a detailed personal analysis of their motor performance changes over time (t0–t2) as an incentive for study participation. As ethical guidelines set by the funding agencies did not permit denying the intervention to any interested individuals who met the inclusion criteria, random allocation was not feasible. Participants were eligible if they were aged 60 years or older and physically inactive, defined as not engaging in regular structured physical or sports activities and not meeting current physical activity recommendations. Individuals were excluded if they regularly participated in structured physical or sports activities, had experienced an acute cardiovascular event (e.g., myocardial infarction or stroke) within the past six months, presented with severe functional limitations affecting mobility, or had unstable or severe physiological conditions contraindicating participation in moderate-intensity physical activity. Everyday activities such as walking, cycling for transportation, housework, or gardening were permitted and did not constitute exclusion criteria. Owing to the program's low-threshold design, no formal exclusion criteria regarding psychosocial impairments were applied, as the group setting aimed to minimize barriers and foster a supportive environment. Primary analyses were conducted per protocol (≥75% attendance) to ensure sufficient intervention exposure.

In addition to obtaining participants’ informed consent, a physician's medical clearance was required. Physical inactivity of all participants was verified in advance by telephone interview and using the Physical Activity and Sport Activity Questionnaire (BSA-F) (33). The focus of physical activity assessment was placed on the regularity and type of participation in organized sports or training groups within clubs, which was not permitted. Everyday activities such as housework, gardening, walking, or cycling to work or for shopping (less than 150 min moderate activity per week) were not considered exclusion criteria.

An *a priori* power analysis was conducted using G*Power (Version 3.1.9.7) for a mixed-design ANOVA with repeated measures (2 × 3 design: two groups over three time points; *α* = .05, 1–*β* = .95, effect size f = .25), yielding a required total sample size of 44 participants. For the analysis of performance changes, the final sample comprised 133 participants: 101 in the IG and 32 in the CG. The group imbalance was due to the ethical obligation to offer the intervention to all interested eligible older adults, precluding strict matching or size restrictions.

### 2.3 Exercise program

Participants of the IG attended two 90-minute exercise sessions per week in fixed training groups of 15–20 participants, mainly in the gym of the University of Magdeburg. The 24-week program consisted of two complementary components: 50% comprised a combined multicomponent fitness training session led by qualified project staff, and 50% a second session in which instructors from local sports clubs in Magdeburg provided introduction classes in various health and recreational sports.

A total of 16 new sports groups for previously inactive older adults were launched on a quarterly basis. Although the intended group size was 15–20 participants, actual enrollment varied considerably across groups, and not all groups reached full capacity at all times. Therefore, despite the planned group size, the total number of recruited participants in the intervention group amounted to 226. The number of initiated groups reflects the program structure rather than a fully saturated group capacity. The program started with low-intensity workloads that were progressively increased over the intervention period by extending the duration and intensity of strength and endurance components, increasing repetitions and sets, and reducing rest intervals, while maintaining perceived exertion predominantly within a moderate range.

The training followed a classical session structure addressing strength, endurance, flexibility, and coordination, combined with technical instruction in fundamental elements of several sports (e.g., karate, table tennis, handball, and archery). High-intensity interval training (HIIT) elements and playful exercise formats were incorporated to enhance engagement and maintain a dynamic training atmosphere.

The intervention was designed in accordance with key domains of the TIDieR checklist (e.g., provider, procedures, intensity, duration, and tailoring) (34) to facilitate replication in community settings. Further technical details regarding intervention delivery and cooperation with local sports clubs are described elsewhere (7, 32, 35).

During the final phase of the program, participants received targeted information about local sports clubs that cooperating in the project, support in selecting suitable courses, and, where needed, assistance with contacting clubs and attending further trial sessions to facilitate a smooth transition into regular membership.

### 2.4 Motor tests and outcome parameters

Six different test procedures were employed to assess selected motor skills that are most commonly used to evaluate everyday-relevant functions and mobility in older adults: Hand dynamometer Test (36), Biceps-Curl-Test and 30s-Chair-stand-Test (37), Drop-bar-Test (38), Sit-&-reach-Test (39) and Physical Working Capacity 130 (PWC 130) (40). All of these tests are feasible for untrained older adults, according to their respective test manuals (Table 1). Unlike the other test procedures, the PWC 130 was only performed at t0 and t2 to limit cardiovascular strain and testing burden for older, untrained participants and to focus on pre–post changes in cardiorespiratory fitness.

**Table 1:** Description of motor tests.

Test procedure	Task description	Capability area
Hand dynamometer Test	Three grip trials with the dominant hand [kg]; the best attempt was recorded.	Max. grip strength
Biceps-Curl-Test	Number of biceps curl repetitions [n] performed within 30 s.	Muscular endurance of the upper extremities
30s-Chair-stand-Test	Number of repetitions [n] of standing up from and sitting down on a chair within 30 s.	Muscular endurance of the lower extremities
Drop-bar-Test	Catching a falling bar as quickly as possible [cm]; the best attempt from three trials was recorded.	Motor reaction ability
Sit-&-reach-Test	Forward trunk flexion with extended legs, measuring fingertip reach on a centimeter scale [cm].	Flexibility of the lower body
Physical Working Capacity 130 (PWC 130)	Incremental step test on a cycle ergometer following the standardized WHO protocol (25/25/2). Relative wattage output W/kg was calculated.	Cardiovascular fitness

### 2.5 Psychosocial well-being and sustainability

Data on the sustainability of continued physical activity, choice of sports programs, and psychosocial well-being were collected using a self-developed Sustainability-Questionnaire (S-Q; Supplement 1) four weeks after the project's completion and again at six- and 12-month post-intervention (RI and RII). As the S-Q was specifically designed for this project and its psychometric properties have not yet been formally established, analyses of sustainability and psychosocial outcomes were limited to descriptive and exploratory evaluations. Regarding participants’ regular engagement in sports, quantitative questions addressed the type of sport practiced, membership in one or more sports clubs, and the selection of sports programs. The number of weekly sports sessions/courses attended at a club, along with their duration in minutes, was also recorded. The motives for engaging in sports were based on the Bernese Motive and Goal Inventory (41). Interim results were published in 2024 (7) and will be completed in due course.

In terms of psychosocial well-being, both the relevance of sports for individual well-being and body perception immediately after a completed exercise session were assessed. To provide a longer-term evaluation of the effects of the exercise program on social activity and social environment, participants were asked about their motivation for everyday activities and the perceived external view of their social interactions.

### 2.6 Statistical data analysis

For the analysis of psychosocial well-being and sustainability in the IG, all available data (*n* = 161) were recorded in Excel and processed descriptively. The datasets for RI were also complete, whereas for RII data were available from *n* = 157 participants. Therefore, the results for the sustainability data are reported as relative percentages of the sample.

Statistical analyses were conducted using R (version 4.4.3). For the analysis of longitudinal motor test data, participants were assigned to either the IG or the CG, and within each group further subdivided by sex (male and female). Due to unequal sample sizes, the presence of outliers, and some non-normal distribution (skewness) of the data, the Brunner-Langer model (F2-LD-F1 model; nparLD package) as a nonparametric alternative to repeated-measures mixed ANOVA was applied (42). Main effects (Group, Time) and interaction effects (Group*Time) were evaluated using Wald-type statistics (WTS), ANOVA-type statistics (ATS), and a modified ANOVA-type statistic with Box approximation, all based on rank transformation (43). The normally distributed data of the PWC130 were calculated using mixed ANOVA (two-way repeated measures). Friedman and Wilcoxon tests with Bonferroni–Holm correction were employed as *post hoc* procedures for pairwise comparisons of longitudinal data between IG and CG, as well as between males and females within IG. A significance level of *α* = .05 was adopted. Significant effects identified via the F2-LD-F1 model were interpreted based on the *Δ*RTE (Relative Treatment Effect) as follows: 0.01 = no difference, ≥ 0.10 = small effect, ≥ 0.15 = medium effect, ≥ 0.25 = large effect. For Post-Hoc-tests the correlation coefficient r was used: < 0.3 = small effect, 0.3 ≤ r < 0.5 = medium effect, ≥ 0.5 = large effect.

## 3 Results

This study aims to examine the influence of the multicomponent exercise program on motor performance parameters as well as on psychosocial well-being and long-term exercise sustainability in previously inactive older adults. It also aims to examine how training affects selected motor skills compared to an inactive control group.

### 3.1 Sample

At baseline (t0), 226 older adults were enrolled in the intervention cohort, with 17 participants withdrawing before the intervention began. During the intervention period, an additional 35 participants discontinued due to noncommunicable diseases and eight due to time-related reasons, such as caregiving responsibilities for relatives or grandchildren. Consequently, these 43 participants did not meet the required 75% training attendance rate. After accounting for these withdrawals, 166 participants remained eligible for follow-up assessment. However, five additional participants were excluded from the sustainability questionnaire (S-Q) analysis because they were older than 80 years and represented a very small age subgroup, which was not included in the predefined analytical framework of the S-Q evaluation. Therefore, at the post-test measurement (t2) and S-Q, 161 older adults participated and were fully included in the analysis regarding psychosocial well-being and the sustainability of the exercise program (Table 2).

**Table 2:** Sample characteristics .

Sample	Motor Tests		PWC 130		S-Q
	IG	Total CG	IG	CG	Total IG
	(60–69 years)		(60–69 years)		
Baseline characteristics					
n	101	32	79	23	161
Sex (%)	f: 71 (70%)	f: 22 (69%)	f: 57 (72%)	f: 14 (61%)	f: 107 (66%)
	m: 30 (30%)	m: 10 (31%)	m: 22 (28%)	m: 9 (39%)	m: 54 (34%)
Age (years, M ± SD)	65.39 ± 3.54	65.63 ± 3.63	65.3 ± 2.29	65 ± 2.8	68.23 ± 4.47
Years of physical inactivity	C: 27.7%	C: 21.9%	C: 25.3%	C: 17.4%	C: 24.2%
	>5: 15.8%	>5: 21.9%	>5: 15.9%	>5: 21.7%	>5: 19.2%
	>10: 14.9%	>10: 21.9%	>10: 16.5%	>10: 17.4%	>10: 12.4%
	>20: 19.8%	>20: 15.6%	>20: 21.5%	>20: 17.4%	>20: 21.7%
	Y: 21.8%	Y: 18.8%	Y: 21.5%	Y: 26.9%	Y: 22.6%

**n** sample size. **M** mean. **SD** standard deviation. **f** female. **m** male. **C** since corona-pandemic. **>5, >10, >20** more than 5, 10, 20 years. **Y** since youth.

In the control group (CG), the number of participants decreased from *n* = 35 at t0 to *n* = 32 at t2 due to personal and time-related reasons regardless to the study. For the assessment of motor abilities, only data from IG participants aged 60–69 years at t0–t2 (*n* = 101) were included in order to match them to the CG on the basis of age.

For the performance comparison of cardiovascular fitness in the PWC 130 test, only a smaller subset of data could be used (IG: *n* = 79; CG: *n* = 23), as not all participants were able to complete the endurance test at either the pre- or post-test measurement. In the CG, nine participants were excluded because they completed the test at t0 but not at t2 (target heart rate < 130 bpm). In the IG, data from 22 participants were excluded: 11 participants did not reach 130 bpm at either t0 or t2; three participants achieved 130 bpm at the pre-test but were unable to exceed 130 bpm at the post-test due to muscular limitations despite being at a higher workload; and eight participants only completed the step test at the post-test due to their initial fitness status.

The *χ*^2^ test revealed no significant differences between the samples of the motor tests - IG (60–69 years) and total CG - with respect to sex (*p* = .868) and age distribution (*p* = .092), nor between the samples of the PWC 130 - IG (60–69 years) and CG - for sex (*p* = .301) or age (*p* = .895).

### 3.2 Comparison of motor abilities between the intervention group (IG) and the control group (CG)

The means and standard deviations for all measurements are shown in Table 3. At baseline (t0), no statistically significant differences were found between the IG and CG in motor parameters (hand dynamometer test, biceps-curl-test, 30s-chair-stand-test, drop-bar-test, sit-&-reach-test and PWC 130 (*p* > .05 for all).

**Table 3:** Results of motor performance from baseline (t0), intermediate (t1) and post-test (t2) for the intervention group and control group.

Test procedure	Intervention group *n* = 101			Control group *n* = 32		
	t0 M ± SD	t1 M ± SD	t2 M ± SD	t0 M ± SD	t1 M ± SD	t2 M ± SD
Hand dynamometer Test [kg] ↑	29.72 ± 8.8	29.7 ± 9.38	28.93 ± 8.9	31.9 ± 8.83	31.58 ± 8.4	32.64 ± 9.6
Biceps-Curl-Test [n] ↑	23.85 ± 4.6	25.9 ± 5.28	26.4 ± 5.05	25.69 ± 5.3	26.78 ± 5.7	26.06 ± 5.1
30s-Chair-stand-Test [n] ↑	16.9 ± 4.4	18.7 ± 5.09	19.9 ± 5.18	18.8 ± 4.47	19.87 ± 5.5	19.9 ± 5.18
Drop-bar-Test [cm] ↓	18.8 ± 7.04	17.7 ± 6.52	17.1 ± 6.84	20.63 ± 8.2	18.6 ± 6.79	18.94 ± 5.6
Sit-&-reach-Test [cm] ↑	−3.3 ± 10.7	−1.23 ± 9.46	−1.91 ± 10	−3.97 ± 8.4	−2.72 ± 7.94	−4.06 ± 8.7
	Intervention group *n* = 79			Control group *n* = 23		
PWC 130 [W/kg] ↑	1.13 ± 0.36	-	1.2 ± 0.37	1.02 ± 0.38	-	1.04 ± 0.29

**↑** and **↓** indicate whether a high or low test score reflects optimal performance **M** mean, **SD** standard deviation.

Subsequently, the results were analyzed using the Brunner-Langer model (PWC 130 with mixed ANOVA), regarding the interaction effect between the groups (time*group). For a better interpretation of the interaction effect, the within-group effects were additionally analyzed (Table 4).

**Table 4:** Results of the comparison of the interaction and within effects of the intervention (IG) and control (CG) group.

Test procedure	Time*group effects			Within-group effects				
	*χ*^2^ (df)	*p*	Effect size *Δ*RTE		n	z	p	Effect size r
		(t0, t1, t2)						
Hand dynamometer Test [kg] ↑	4.082 (1.59)	.025	.046	IG CG	101 32	4.493 0.143	.106.931	.03.02
Biceps-Curl-Test [n] ↑	1.12 (1.98)	.324	.091	IG CG	101 32	23.894 2.643	. < .001^a^^,^^b^.267	.06.06
30s-Chair-stand-Test [n] ↑	5.127 (1.84)	.007	.150	IG CG	101 32	68.283 2.33	< .001^a^^,^^b^^,^^c^ .311	.11.06
Drop-bar-Test [cm] ↓	0.353 (1.99)	.702	.066	IG CG	101 32	6.414 0.587	.040^b^ .746	.03.03
Sit-&-reach-Test [cm] ↑	0.215 (1.39)	.723	.021	IG CG	101 32	7.115 1.010	.029^a^^,^^b^ .604	.03.04
	F(df)	*p* (t0-t2)	*η* ^2^			*M_Diff_*	*p* (t0-t2)	ƒ
PWC 130 [W/kg] ↑*	0.927 (1,100)	.338	.009	IG CG	79 23	0.083 0.014	.017.827	.25 [CI -,151; -,015].00 [CI -,140;,112]

**↑** and **↓** indicate whether a high or low test score reflects optimal performance. *Mixed Anova. **χ^2^** Test statistic of the WTS. *Δ***RTE** Relative Treatment Effect. **z** Test statistic of Friedman test with df = 2. **r** Pearson's r.

^a^ Within-group effects: significantly different between t0-t1 (statistically significant: *p* ≤ .05).

^b^ Within-group effects: significantly different between t0-t2 (statistically significant: *p* ≤ .05).

^c^ Within-group effects: significantly different between t1-t2 (statistically significant: *p* ≤ .05).

**Figure 2:**
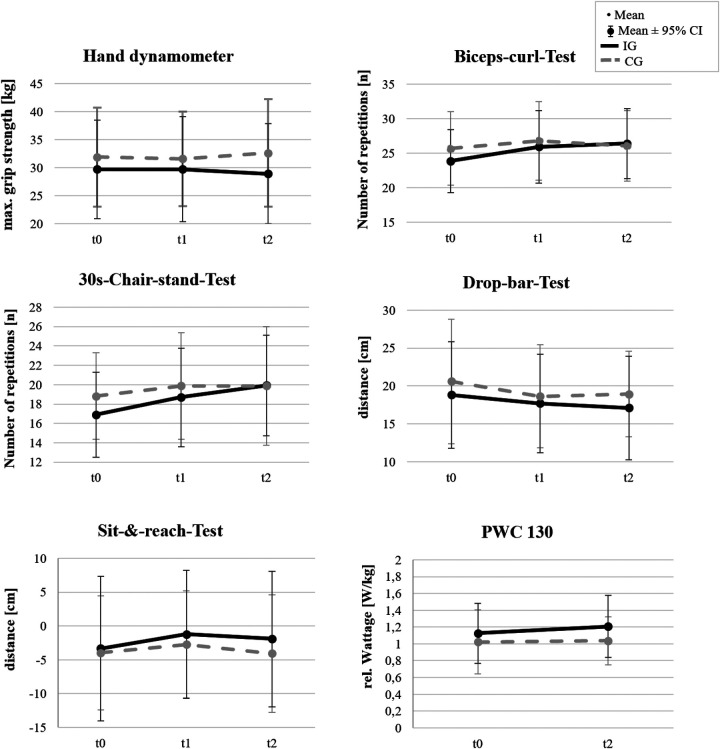
Time*group effects for the motor parameters of IG and CG. Higher scores signal better performance. Only in the Drop bar test the lower value indicates a better performance.

The analyses of the motor performance tests revealed differentiated findings with respect to time, group, and interaction effects with small to moderate effect sizes as well as sex differences within the intervention group (IG). For clarity, the interactions between the IG and CG were visualized in Figure 2.

In the hand dynamometer test, a significant main effect of group was observed in favor of the control group (CG), as the IG showed lower values overall [*χ*^2^(1) = 6.124, *p* = .013, *Δ*RTE = –.116]. In addition, a significant Time*Group interaction was found, whereas no isolated time effect emerged. This indicates a tendency toward decreasing values over time in the IG. Within the IG, men achieved significantly higher values than women (Table 5), with a strong group effect between sexes [*χ*^2^(1) = 131.38, *p* < .001, *Δ*RTE = .41]. The results indicate a slight decrease in men and relatively stable values in women; no interaction effect was observed, and neither gender exhibited a significant change in maximum handgrip strength.

**Table 5:** Results of the comparison of the interaction and within effects of males and females (IG).

Test procedure	Time*group effects			Within-group effects				
	χ^2^ (df)	*p*	Effect size *Δ*RTE		n	z	*p*	Effect size r
		(t0, t1, t2)						
Hand dynamometer Test [kg] ↑	0.429 (1.59)	.605	.41	m f	30 71	0.600 4.745	.643 .086	.040 .040
Biceps-Curl Test [n] ↑	0.32 (1.98)	.746	.01	m f	30 71	10.661 13.705	.021^a^^,^^b^ .005^a^^,^^b^	.14 .07
30s-Chair-stand Test [n] ↑	1.06 (1.84)	.339	.08	M f	30 71	18.054 50.847	< .001^b^^,^^c^ .004^a^^,^^b^^,^^c^	.19 .14
Drop-bar Test [cm] ↓	0.123 (1.99)	.884	.02	m f	30 71	6.50 1.919	.054.110	.12.03
Sit-&-reach Test [cm] ↑	2.041 (1)	.145	.30	m f	30 71	6.278 3.000	.002^a^ .028^b^	.11.03
	F(df)	*p* (t0-t2)	*η* ^2^			*M_Diff_*	*p* (t0-t2)	ƒ
PWC 130 [W/kg] ↑*	.096 (1,77)	.758	.001	m f	22 57	.067.090	.290.024	.12 [CI −0.192;0.058].11 [CI −0.167; −0.012]

**↑** and **↓** indicate whether a high or low test score reflects optimal performance, *Mixed Anova, **χ^2^** Test statistic of the WTS, ***p*-value**, *Δ***RTE** Relative Treatment Effect, **z** Test statistic of Friedman test with df = 2.

^a^ Within-group effects: significantly different between t0-t1 (statistically significant: *p* ≤ .05).

^b^ Within-group effects: significantly different between t0-t2 (statistically significant: *p* ≤ .05).

^c^ Within-group effects: significantly different between t1-t2 (statistically significant: *p* ≤ .05).

In the biceps curl test, no group or interaction effects were observed [*χ*^2^(1) = 0.38, *p* = .539, *Δ*RTE = .030]. However, a significant main effect of time was identified [*χ*^2^(1.98) = 8.27, *p* < .001, *Δ*RTE = .030], indicating performance improvements across measurements. The pairwise *post hoc* comparison revealed a significant performance increase only in the IG between measurements t0–t1 and t0–t2, whereas no significant changes were observed in the CG. Within the IG, no overall Time*group effects (with group referring to sex) were found, but *post hoc* analyses revealed significant improvements over time with small effects in both sexes in t0-t1 and t0-t2.

The chair stand test also revealed a significant effect of time [*χ*^2^(1.84) = 13.344, *p* < .001, *Δ*RTE = .137] and an moderate significant effect for time*group, but no group effects. Within the IG, both men and women significantly improved their performance, with women showing stronger effects across all time points (t0-t1, t0-t2 & t1-t2). Men improved significantly after 3 months of exercise between t0–t2 and t1–t2. No significant changes were observed in the CG.

In the drop-bar test, no group, time, or interaction effects were detected (*p* > .05). Within-group effects shows only significant improvements for the IG from t0 to t2. Likewise, within the IG, no significant sex differences were found.

The sit-and-reach test did not yield significant main or interaction effects for group or time (*p* > .05), but a significant within-group effect of the IG between t0-t1 & t0-t2. However, significant sex differences were found within the IG, with women achieving higher flexibility values than men [*χ*^2^(1) = 11.615, *p* < .001, *Δ*RTE = .30]. Both sexes, however, maintained stable performance across time.

For the PWC130 ergometry test, no significant main effect of time was observed [F(1,100) = 1.819, *p* = .180, *η*^2^ = .018]. However, a significant group*time interaction was detected, with the IG demonstrating significant improvements with a moderate effect, while the CG remained unchanged. Within the IG, sex differences emerged [*χ*^2^(1,77) = 4.845, *p* = .031, *Δ*RTE = .059], but no time*group interaction effect was found. However, pairwise *post-hoc* comparisons indicated that women achieved significantly larger improvements than men.

### 3.3 Change in psychosocial well-being through physical activity

Subjective and psychosocial well-being in relation to the exercise program was assessed using the Sustainability Questionnaire (S-Q). As shown in Table 6, the majority of participants rated physical activity as quite or very important for their well-being, reported feeling more energized and recovered after exercise, and perceived positive changes in how their social environment viewed them.

**Table 6:** Results of subjective and psychosocial well-being.

(1) How important is physical activity for your well-being?						
answers	Not at all	A little bit	Moderately	Quite important	Very important	
sample						
total	0%	2%	4%	66%	28% (both sexes)	
*m*		0%	7%	65%		
*w*		2%	2%	67%		
(2) How do you feel after physical activity?						
Answers	Stressed	Tired	Unchanged	Exhausted	Energized	Recovered
Sample						
Total	0%	1%	9%	14%	37%	40%
m		0%	15%	4%	28%	52%
w		1%	6%	20%	41%	34%
(3) Do you feel that your social or family environment perceives you differently?						
Answers	Yes, more positive	Yes, more motivated	Yes, fitter	Yes, something different…	No change	
Sample						
Total	19%	17%	42%	6%	30%	
m	17%	15%	46%	6%	31%	
w	21%	18%	39%	6%	29%	
(4) Do you feel more motivated today, beyond your regular exercise, to engage in activities or outings?						
Answers	Yes, more cultural events	Yes, more sport	Yes, more social gatherings	Yes, but lack of time	No change in motivation	No, less motivation than before
Sample						
Total	38%	38%	40%	2%	29%	1%
m	33%	37%	46%	2%	35%	0%
w	40%	38%	36%	3%	25%	1%

Multiple answers possible for questions (3) and (4) (S-Q; *n* = 161).

### 3.4 Sustainability of the exercise program

**Figure 3:**
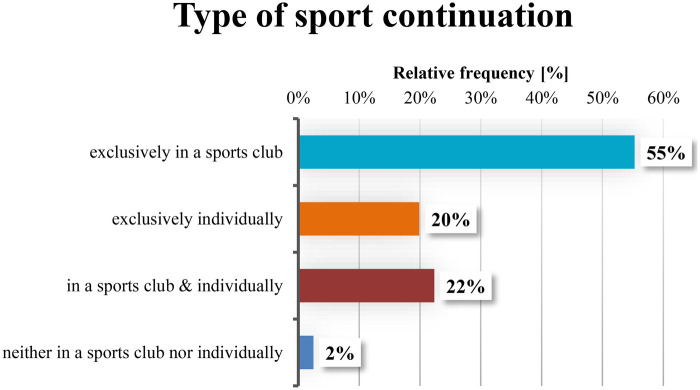
Continuation of regular physical activity four weeks after project completion (S-Q; *n* = 161).

As already indicated in the previous work (7), a high transition rate of study participants into regular sports activity was observed following completion of the project. Approximately 97% (*n* = 125) of the 161 participants remained regularly physically active after the exercise program (Figure 3). The gender differences were minor in the ratio between club members (w = 77%, m = 80%) and exclusively self-organized sporting activity (w = 23%, m = 20%). Of these 161 participants, a total of 77% of the older adults had joined either one (50% of participants) or multiple (27% of participants) local sports clubs. Data from Retention Tests I (RI) and II (RII) show that up to one year after the end of the project, the proportion of participants holding club memberships had increased to 87%. In addition, participants who had previously engaged in sports exclusively on their own had, by this time, joined a training group (S-Q = 20%; RII = 13%).

**Figure 4:**
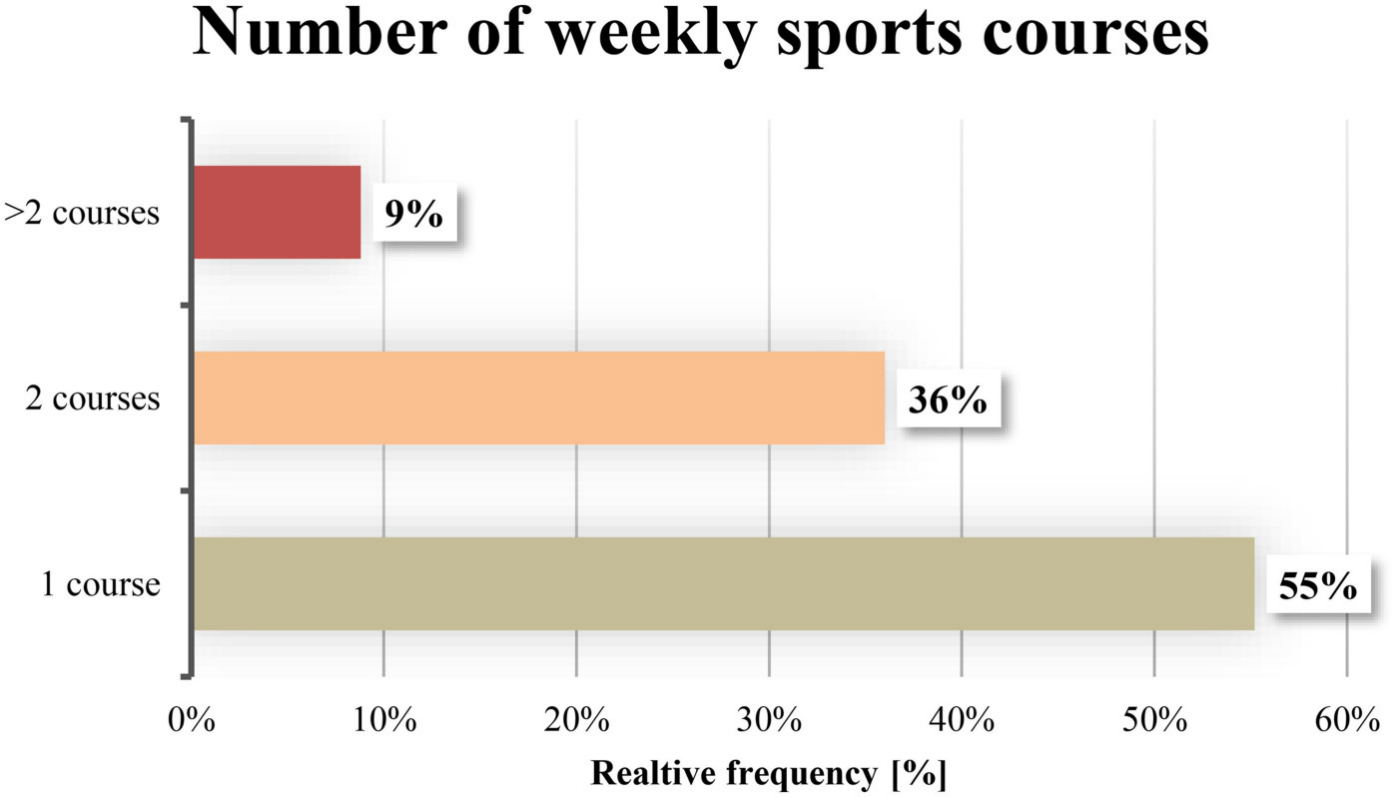
Participation in regular weekly sports sessions after project completion (S-Q; *n* = 125; w = 82, m = 43).

In addition to joining multiple sports clubs (S-Q = 27%; RII = 37%), the number of regular weekly sports courses (Figure 4) attended within clubs also increased after one year: 33% participated in one course per week, 39% in two courses, and 12% of club members engaged in more than two courses per week. Overall, participants - with or without club membership - achieved an average of 201 min of vigorous physical activity per week, thereby meeting the WHO recommendations for physical activity.

**Figure 5:**
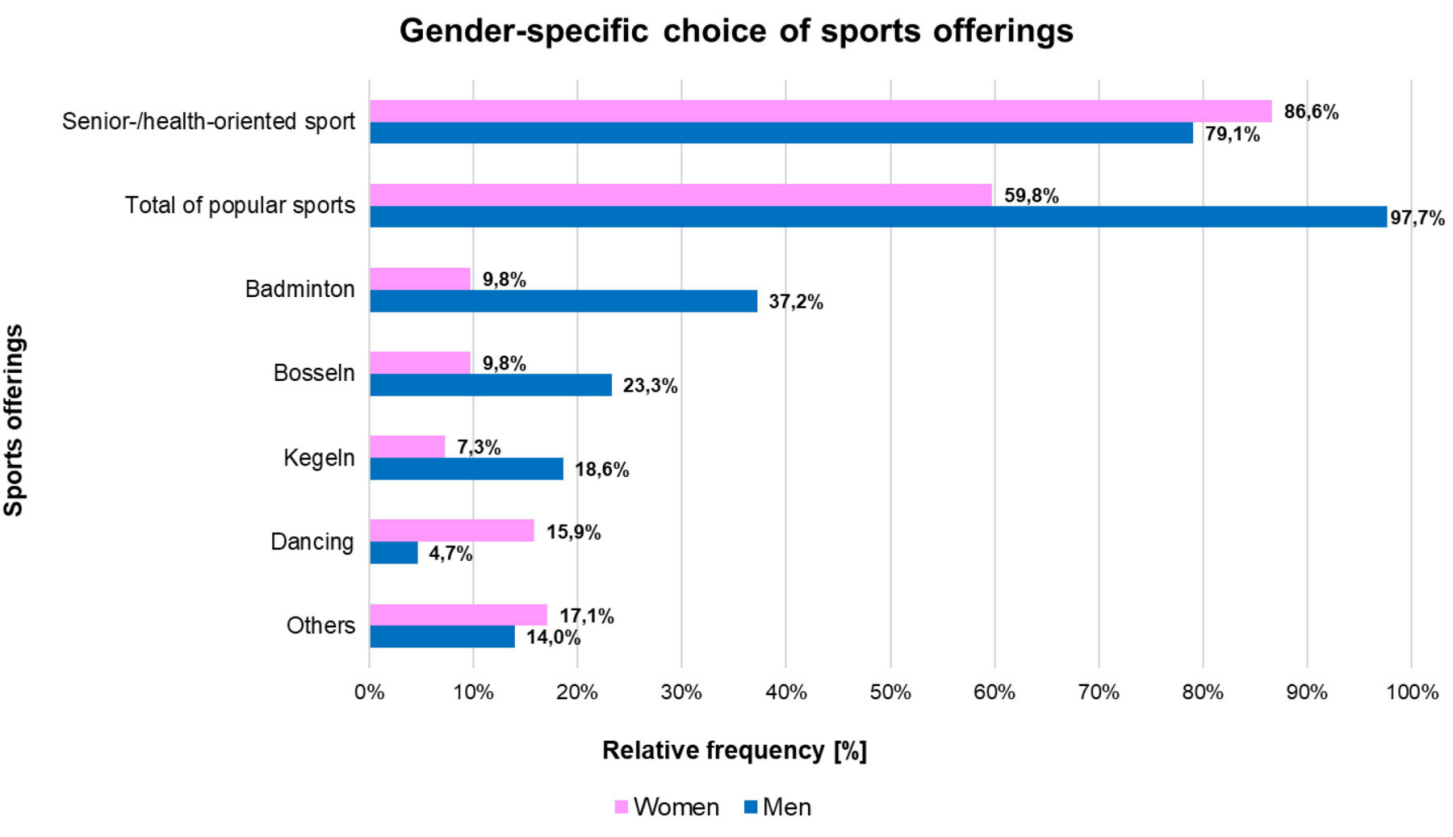
Choice of sports offerings as gender-specific relative frequency after completion of the exercise program (*n* = 125; w = 82, m = 43; multiple responses possible).

Regarding the choice of sports offerings, the distribution between men and women changed slightly in the published interim results (*n* = 93) (7): while participants previously showed that approximately 85% of women and 76% of men primarily chose senior or health-oriented sports courses as a form of recreational sport (64% of women and 69% of men), the overall results of the S-Q (Figure 5; *n* = 125) indicate that male participants, in contrast to female participants, were more actively engaged in recreational sports offerings (97.7%) than in senior-specific programs (79,1%). This gender-specific preference for sports offerings remained similar in Retention Tests I and II. The relative percentage distribution of sport program choices, allowing for multiple responses, further indicates that many older adults maintained a combination of age-specific activities together with general recreational sports offerings.

The motives for participating in sports did not fundamentally change compared to the published interim results (7), with the main motives continuing to be “health” (96%), “fitness” (86%), and both “cognition” and “enjoyment of movement” (each 74%).

## 4 Discussion

The present data indicate that the sport-oriented, multicomponent exercise program was associated with selective improvements in motor performance and cardiovascular fitness among previously inactive older adults and also demonstrated high effectiveness with respect to sustainability. Significant gains over time, with a medium interaction effect, were observed in the chair stand test, while smaller within-group improvements occurred in the biceps curl, drop-bar, sit-and-reach, and PWC130 tests. Unexpectedly, the maximum grip strength of the intervention group decreased slightly, particularly among men, though this was not statistically significant. Both sexes benefited, with women exhibiting somewhat greater relative improvements, particularly in the PWC130.

These findings align well with existing literature on multicomponent training. Systematic reviews and meta-analyses report that combining strength, endurance, coordination, and balance exercises yields robust improvements in muscular strength, mobility, and sometimes cardiovascular fitness in older adults (44–46). The improvements in lower-limb strength (chair stand) are consistent with evidence that resistance and functional training substantially increase leg strength and daily functional capacity (47). Gains in the PWC130 likewise correspond to findings that aerobic and combined endurance–resistance training enhances cardiorespiratory fitness, with effects depending on intensity and volume. Meta-analyses further show that both moderate endurance training and HIIT produce meaningful increases in VO₂ max, with multicomponent approaches combining these effects advantageously (48, 49).

That handgrip strength improved only marginally is not surprising, as maximal strength measures largely reflect absolute strength levels and depend on baseline status, training intensity, and specificity. When interpreting the magnitude of functional improvements, it should be considered that approximately half of the weekly training volume consisted of introductory sessions to different recreational sports. In these sessions, the focus was often on learning basic techniques and reducing perceived barriers rather than maximizing training intensity, which may partly explain why gains in maximal strength were modest while functional and endurance-related outcomes improved. Short-term, broadly designed programs can elicit functional improvements without immediate increases in maximal strength, which typically require more targeted progressive resistance training. This distinction between functional and maximal strength outcomes has also been described previously (47).

In addition to gains in motor performance, participants also reported improvements in psychosocial well-being. Based on the exploratory and descriptive data obtained from the self-developed Sustainability Questionnaire, participants indicated increased engagement in social and cultural activities and perceived greater social recognition. Many expressed an awareness that regular participation in sport had become a key contributor to their overall sense of well-being. These results are in alignment with evidence indicating that multicomponent, community-based interventions can simultaneously enhance physical activity levels and social participation among older adults (5, 25). In line with existing studies, structured multicomponent exercise has been shown to improve quality of life and biopsychosocial health - including mood, social functioning, and mental health - in older populations (26, 50). However, given the non-controlled and exploratory nature of these outcomes, causal conclusions should be drawn with caution.

### 4.1 Sex differences

The sex-specific differences observed within the IG (relative advantages for women in the PWC130 and generally more pronounced gains in women) are consistent with the current literature: recent reviews (17, 51) highlight sex-related variations in training adaptations - men tend to achieve greater absolute increases in maximal strength, whereas women demonstrate comparatively larger relative improvements from baseline in certain endurance and functional parameters. The underlying physiological mechanisms are multifactorial, including differences in muscle composition, hormonal profiles, metabolism, and recovery capacity. These findings underscore the relevance of sex-specific program design and dose adjustment (51).

### 4.2 Sustainability

A particularly noteworthy finding is the high sustainability of the program, with 97% of participants continuing to exercise regularly and approximately 87% transitioning into local sports clubs one year after completing the project. Its low-threshold design with homogeneous intervention groups, diverse recreational options, and gender-sensitive structure, facilitated long-term adherence and help address the “prevention dilemma”, whereby preventive programs often primarily reach individuals who are already relatively active. The gender-sensitive structure included the targeted provision of sport types perceived as attractive by both sexes (e.g., ball games, martial arts, and table tennis), scheduling sessions at times convenient for retirees with caregiving responsibilities, and fostering a non-stigmatizing, performance-oriented yet supportive group atmosphere (8, 11, 12). However, sustainability outcomes were assessed descriptively using a self-developed questionnaire and without a control group; therefore, these results should be interpreted as indicative rather than confirmatory.

The exercise program not only had positive effects on the participants themselves but also benefited local sports clubs, which were able to attract new members through the established cooperation. Owing to the high and continuing demand among older adults and the increase in club membership, the regional city sports federation of Magdeburg has institutionalized the program on a permanent basis and will continue its implementation in the near future.

### 4.3 Limitations

The study has several key limitations that should be considered when interpreting and generalizing the findings. First, the absence of randomization and the substantially smaller control group introduce risks of selection bias. Second, the voluntary nature of participation and the per-protocol analytical approach raise the possibility of self-selection and motivation biases. Specifically, 43 participants (19%) did not reach the predefined 75% attendance rate and were therefore excluded from the analyses, which may have led to an overestimation of intervention effects. Third, participant attrition between measurement points reduced sample size and may have further biased results. Moreover, training intensity of the local sports clubs was not strictly standardized across all sessions, so between-group or within-group differences in dose could have influenced the magnitude of observed effects. A sex imbalance was observed (intervention group: 148 women vs. 78 men). Considering that participation rates of older men in preventive exercise programs are consistently reported to be substantially below those of women, the inclusion of 78 previously inactive older men represents a meaningful achievement of the program's targeted recruitment strategy, as consistently reported in public health research (8, 13, 14). Finally, sustainability and psychosocial outcomes were assessed using a self-developed questionnaire whose validity and reliability have not yet been formally evaluated; future studies should therefore include systematic psychometric validation. Future research would benefit from randomized designs, larger and better-matched control groups, and more precise quantification of training dose (e.g., detailed load protocols and objective activity monitoring).

### 4.4 Conclusion

In summary, the 24-week multidimensional exercise program appears to be a practical and potentially model for promoting physical activity among older, previously inactive adults. It produced modest functional fitness, most notably in lower-limb strength, with smaller within-group gains in biceps curl, flexibility and drop-bar performance. Cardiorespiratory fitness (PWC130) improved in the intervention group, and women showed larger gains than men. Handgrip strength tended to decline in men and was overall lower in the IG.

The low-threshold exercise program facilitates (re-)entry into physical activity and integration into the local sports infrastructure, demonstrating high sustainability (97% of participants remained active; 87% joined local clubs after one year). Gender-specific differences were evident in the manner of continued participation, and the program also enhanced psychosocial well-being. Recognizing its benefits for both older adults and local sports clubs, the City Sports Federation of Magdeburg has permanently institutionalized the concept. These promising signals warrant further investigation in properly powered randomized trials to confirm long-term effectiveness and cost-effectiveness prior to widespread implementation.

## Data Availability

The raw data supporting the conclusions of this article will be made available by the authors, without undue reservation.
