# Supplementary material for: Influence of a sport-oriented exercise concept on motor performance and sustainability in healthy but sporting inactive older adults aged 60+
Source: Front Sports Act Living. 2026 Mar 25;8:1702331. doi: 10.3389/fspor.2026.1702331 (PMC13057376; doi:10.3389/fspor.2026.1702331)
Supplement: Supplementary file 1 [file Datasheet1.pdf]

Sustainability Questionnaire

Question 1: Activity in sports clubs/foundations

**1.a**	Yes, in more than one	Yes	No
Have you become a member of one or more sports clubs?	☐	☐	☐

→ If you answered 'yes' or 'in more than one,' please continue with **Question 2.**

**1.b**	Yes	No
Do you regularly do sports without a club?	☐	☐

1.c Which sport(s) do you practice?

____

1.d How many minutes per week do you do sports?

☐ Cycling Min:

☐ Running Min:

☐ Swimming Min:

☐ Other activity: _________________ Min:

1.e Why did you not join a sports club?

☐ I did not find a suitable offer for me

☐ I am still undecided where I want to become a member

☐ I have not found anyone to go with, and I don't want to go alone

☐ I missed the direct transition after the university project

☐ I lack motivation

☐ other:

Question 2: Activity through club offers

2.a In which club(s) did you become a member/are you active in a foundation?

Club: ____________________________________

Club: ____________________________________

Club: ____________________________________

2.b Did you get to know the club/sports offer through the trial sessions in the project?

☐ Yes

☐ No

☐ The club is known to me through the project, but I do other sports offers there

☐ The sports offer is known to me from the trial sessions, but I chose a different club

→ If you selected the last option, please continue with **Question 2c**

2c. Why did you choose another club?

☐ Closer to my home

☐ I preferred the atmosphere in the course

☐ The club made a better impression on me

2.d weekly activity

	One course	Two courses	> 2 courses
How many sports courses do you attend regularly?	☐	☐	☐

2.e How many minutes per week do you train regularly in the sports club?

____

2.f Which sports offers/courses do you attend? (multiple answers possible)

☐ Senior sports/health sports/gymnastics

☐ Bosseln

☐ Dancing

☐ Other: ________________________

☐ Badminton/Federball

☐ Kegeln/Bowling

☐ Billards

☐ Yoga

2.g Do you also occasionally engage in irregular sports activities?

☐ no ☐ yes, namely

☐ Kegeln/Bowling ☐ Swimming
☐ Federball ☐ Billards
☐ Bosseln ☐ Other: ______________________

Question 3: Importance of sports activity

**3.a**	Not at all	A little	Moderately	Quite important	Very important
How important is physical activity for you?	☐	☐	☐	☐	☐

3.b For what reasons do you participate in sports activities? (multiple answers possible)

☐ Group dynamics
☐ Routine
☐ Social contacts through sport
☐ Appearance/figure
☐ Mood regulation
☐ Fitness (especially to stay fit)
☐ Health (to maintain independence in daily life)
☐ Aesthetics (because sport gives me the chance to move beautifully)
☐ Preservation of cognitive functioning
☐ Enjoyment of movement
☐ new challenge
☐ Competition/performance

3.c How do you usually practice sports?

☐ alone
☐ with a partner
☐ in a group

Question 4: Well-being and sports activity

**4.a**	energized	recovered	unchanged	exhausted	tired	stressed
How do you feel after physical activity?	☐	☐	☐	☐	☐	☐

**4.b**	Not at all	A little	Moderately	Quite important	Very important
How important is sports activity for your well-being?	☐	☐	☐	☐	☐

4.c In which motor or cognitive abilities do you feel fitter? (multiple answers possible)

a) Motor skills:

- [ ] Endurance
- [ ] Strength
- [ ] Flexibility
- [ ] Coordination
- [ ] Balance

b) Cognitive skills:

- [ ] Attention
- [ ] Memory
- [ ] Reaction ability

4.d Do you feel that your social or family environment perceives you differently? (multiple answers possible)

- [ ] Yes, more positive
- [ ] Yes, more motivated
- [ ] Yes, fitter
- [ ] Yes, something different…: ______________________________
- [ ] No change

4.e Do you feel more motivated today, beyond your regular exercise, to engage in activities or outings?

- [ ] Yes, more cultural events
- [ ] Yes, more sport
- [ ] Yes, more social gatherings (family/friends)
- [ ] Yes, but lack of time
- [ ] No change in motivation
- [ ] No, less motivation than before
